# Supplementary material for: The macroevolutionary impact of recent and imminent mammal extinctions on Madagascar
Source: Nat Commun. 2023 Jan 10;14:14. doi: 10.1038/s41467-022-35215-3 (PMC9832013; doi:10.1038/s41467-022-35215-3)
Supplement: Supplementary file 1 — Supplementary Information [file 41467_2022_35215_MOESM1_ESM.pdf]

Supplementary Information for:

The macroevolutionary impact of recent and imminent mammal extinctions on Madagascar

Nathan M. Michielsen, Steven M. Goodman, Voahangy Soarimalala, Alexandra A.E. van der Geer, Liliana M. Dávalos, Grace I. Saville, Nathan Upham, Luis Valente

Nature Communications

Includes:
Supplementary Figures S1 - S4
Supplementary Tables S1 - S9
Supplementary References

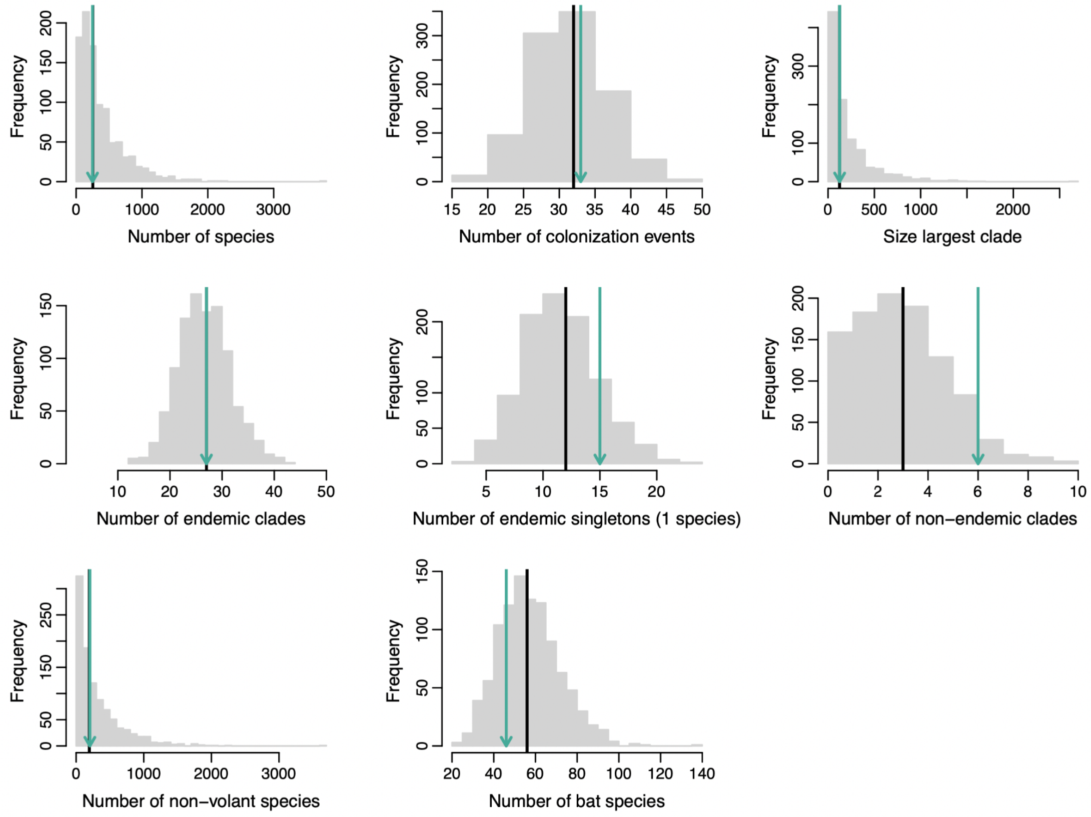

Figure S1 - Goodness of fit of the best overall model, M26. Histograms show the distribution of several diversity metrics across n = 5000 islands simulated under the parameters of the M26 model. Black line - median value of the metric across all simulated datasets. Arrow - empirical value in the Madagascar data. The number of non-endemic clades in the data (6 clades) is different from the number of non-endemic species (9 species) because some non-endemic bats belong to Malagasy radiations and colonized other regions from Madagascar.

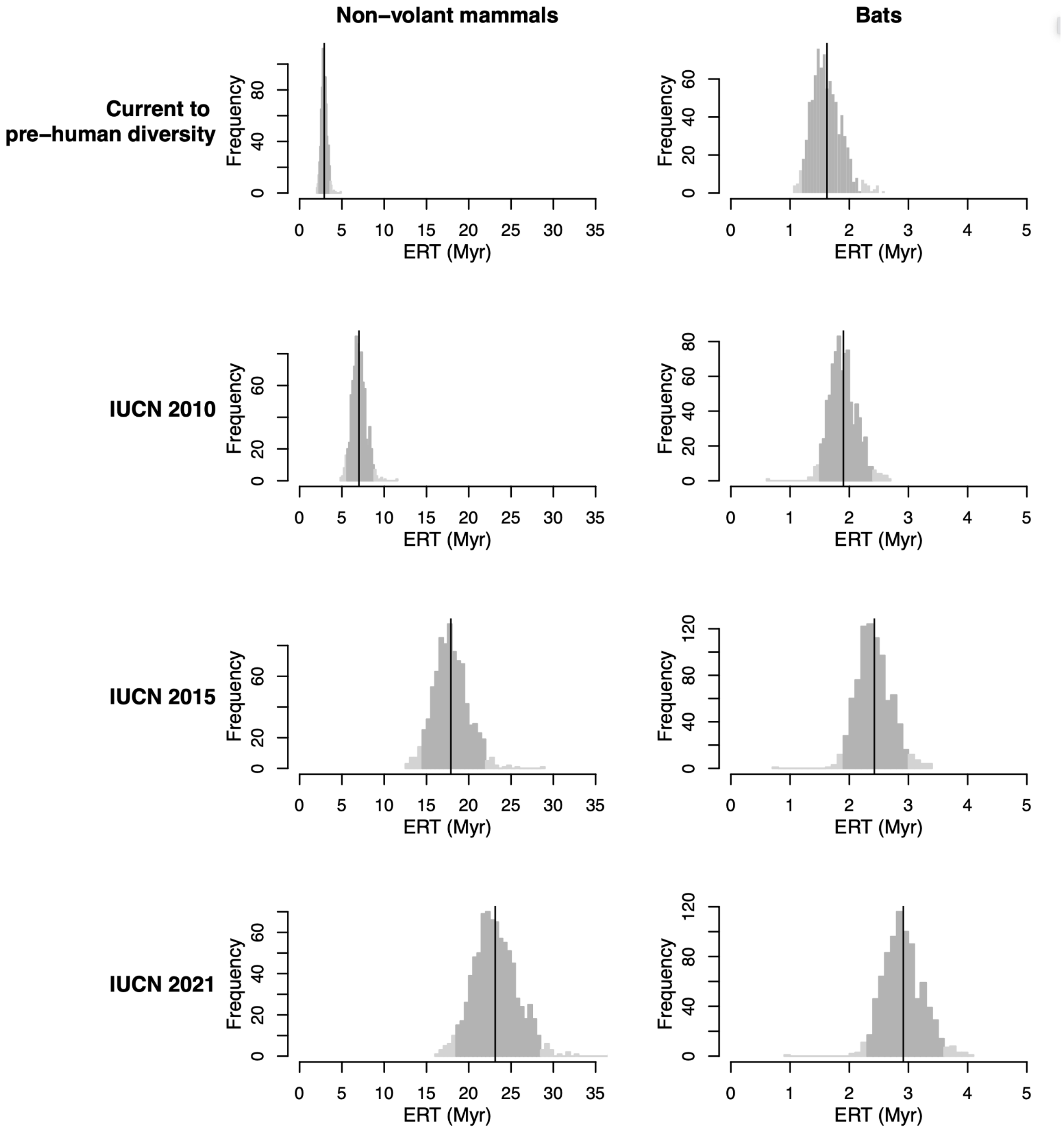

Figure S2 – Histograms of estimated evolutionary return times (ERTs) based on the posterior distribution of trees of the main dataset D1. Distributions of estimated ERTs based on 1000 trees of the posterior are given per diversity target for non-volant mammals and bats. Black lines indicate mean values, and shaded areas indicate the 2.5 – 97.5 percentiles of estimates (n = 1000).

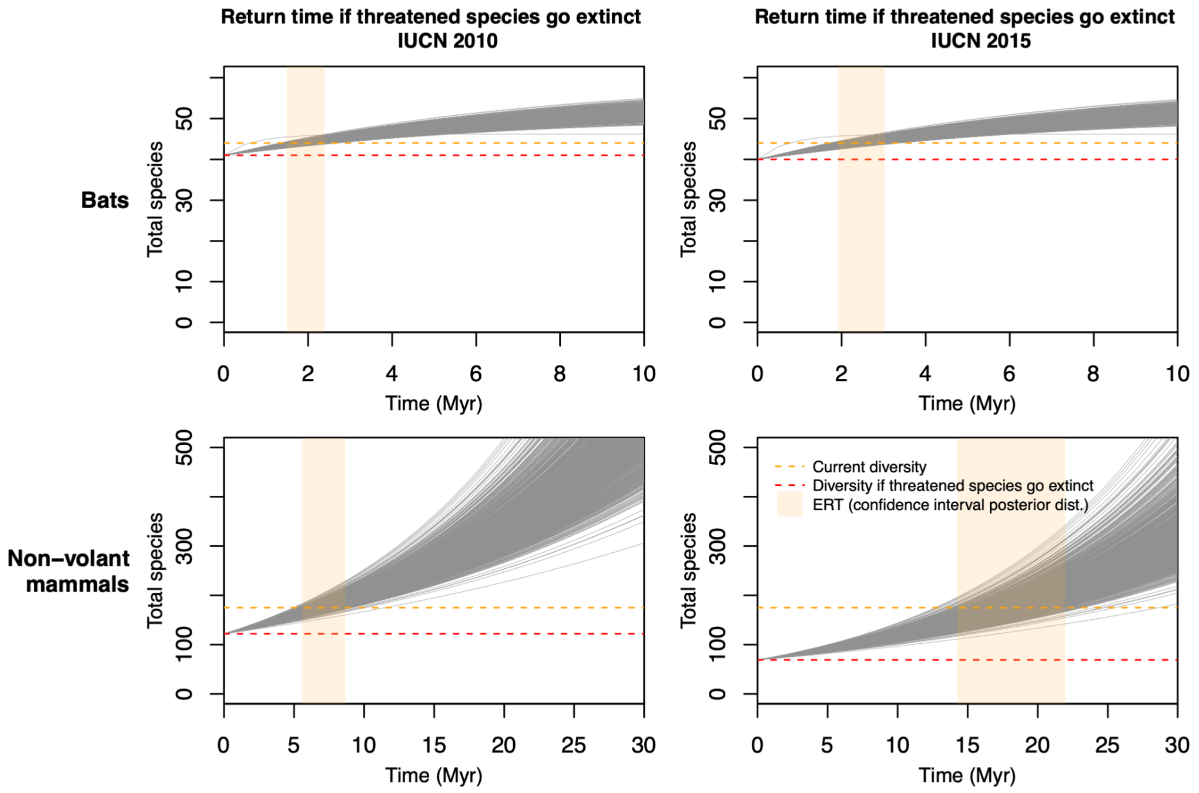

Figure S3 - Expected future diversity for bats and non-volant mammals on Madagascar. Two scenarios are shown: return time to contemporary diversity if species classified as threatened by the IUCN in 2010 go extinct (left panels); and return time to contemporary diversity if species classified as threatened by the IUCN in 2015 go extinct (right panels). Based on fitting the M26 model to 1000 trees from the posterior distribution of the main dataset, D1. The evolutionary return time (ERT) for each tree is the time it takes to go from the start diversity to the target diversity (e.g. red horizontal line to orange horizontal line in the bottom right plot). The vertical shaded area shows the 2.5 - 97.5 percentile of the ERT values based on the posterior distribution of trees (n = 1000).

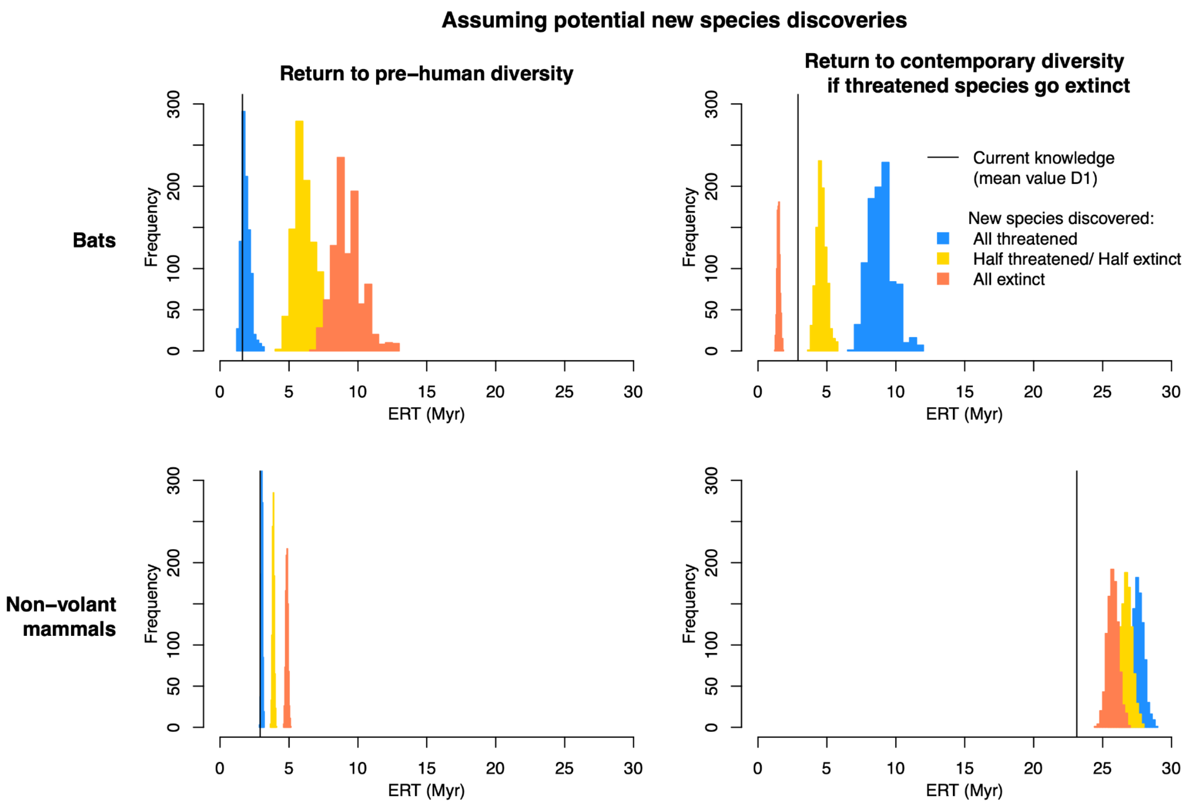

Figure S4 - Effect of future species discoveries on ERT estimates. Assuming 15 new species of bats and 15 new species of non-volant mammals are discovered on Madagascar in the next 10 years. Plots show frequency histograms based on 1000 datasets to which species of bats and non-volant mammals were added at random locations on the phylogenetic data. DAISIE was fitted to each of these datasets, and the ERTs were calculated for each dataset (n = 1000).

Table S1 – The two alternative colonization scenarios (CS) of Madagascar used. C1, C2, etc. are independent colonizations. Numbers in brackets are numbers of species in each colonization. For all groups not listed in the table, a single scenario was supported by the phylogenetic data. See Supplementary Data S2 and S3 for the full data.

CS1	CS2	Justification
C1: Malagasy Afrosoricida (34 sp.)	C1: Tenrecidae (32 sp.)<br>C2: Bibymalagasia (2 sp.)	Bibymalagasia belongs to Afrosoricida and possibly Tenrecoidea, but it is unclear whether it results from the same colonization as the Tenrecidae[81,82]. In CS2, we assume Bibymalagasia is a separate colonisation from the tenrecs, and we used the age of the entire Afrosoricida as a maximum colonization time for Bibymalagasia, to account for uncertainty regarding its precise placement within Afrosoricida.
C1: *Hippopotamus* (3 sp.)	C1: *H. madagascariensis* + *H. lemerlei* (2 sp.)<br>C2: *H. laloumena* (1 sp.)	Some evidence[34] suggests that *H. laloumena* may be the product of separate colonization.
C1: *Macronycteris cryptovalorona* (1 sp.)<br>C2: *M. besaoka* + *M. commersoni* (2 sp.)	C1: *M. cryptovalorona* (1 sp.)<br>C2: *M. besaoka* (1 sp.)<br>C3: *M. commersoni* (1 sp.)	Placement of the extinct species *Macronycteris besaoka* is unclear as there is no molecular data for that species.
C1: *Miniopterus* (12 sp.)	C1: *Miniopterus* 'core' (9 sp.)<br>C2: *M. griveaudi* (1 sp.)<br>C3: *M. mahafaliensis* (1 sp.)<br>C4: *M. sororculus* (1 sp.)	The genus *Miniopterus* on Madagascar is poorly resolved. We assumed a single colonization in CS1 (radiation of 12 species). In CS2 we allowed for 3 additional colonizations, corresponding to the 3 taxa which in the recent phylogeny[83] fall outside the core Malagasy clade.

Table S2 – **Description of the 30 DAISIE models fitted to the phylogenetic data**. Models M1-M4 are homogeneous rates models that assume equal rates across all lineages. Model M5-M30 are 'two-rate' rate models, where bats differ from non-volant mammals in one or more parameters. Gray cells indicate that the parameter is estimated for the model. White cells indicate that the parameter was fixed to a certain value shown in the cell, or, in the case of bats, that it is the same as for non-volant mammals. Diversity-dependent models are those where *K* is estimated, diversity-independent are those where *K* is fixed to infinity. Parameters: λ^c - rate of cladogenesis, μ - rate of extinction, *K* - carrying capacity. γ - rate of colonization, λ^a - rate of anagenesis.

**Model**	λ^c	μ	K	γ	λ^a
M1					
M2			Inf		
M3					0
M4			Inf		0

	**Non-volant mammals**					**Bats**				
	λ^c	μ	K	γ	λ^a	λ^c	μ	K	γ	λ^a
M5										
M6			Inf							
M7								Inf		
M8			Inf							
M9										
M10										
M11			Inf							
M12					0					
M13			Inf		0					
M14					0			Inf		
M15			Inf		0					
M16					0					
M17					0					
M18			Inf		0					
M19										
M20										
M21			Inf							
M22			Inf							
M23										
M24										
M25			Inf							
M26			Inf							
M27										
M28										
M29			Inf							
M30			Inf							

Table S3 - **Maximum likelihood parameters estimated for the main dataset (D1).** Gray cells indicate that the parameter is estimated for the model. White cells indicate that the parameter was fixed to a certain value (shown in the cell), or, in the case of bats, that it is the same as for non-volant mammals. Parameters: λ^c - rate of cladogenesis, μ - rate of extinction, K - carrying capacity. γ - rate of colonization, λ^a - rate of anagenesis. Loglik - log-likelihood. AIC - Akaike Information Criterion. BIC - Bayesian Information Criterion. Npars - number of parameters. *Preferred model for this dataset using BIC or AIC. M1-M4 - homogenous-rate models (same rates for bats and non-volant mammals); M5-M30 - models with different rates for bats and non-volant mammals; M31-M32 - time-variable shift models, with a shift in rate of colonization.

Model	λ^c	μ	K	γ	λ^a	Loglik	AIC	BIC	Npars
M1	0.407	0.418	∞	0.00426	1.438	-648.83	1307.66	1341.39	5
M2	0.407	0.418	∞	0.00426	1.438	-648.83	1305.66	1332.65	4
M3	0.507	0.523	∞	0.00523	0	-660.17	1328.33	1355.31	4
M4	0.507	0.523	∞	0.00524	0	-660.17	1326.33	1346.57	3

	Non-volant mammals					Bats								
Model	λ^c	μ	K	γ	λ^a	λ^c	μ	K	γ	λ^a	Loglik	AIC	BIC	Npars
M5	0.406	0.418	∞	0.00083	1.429				0.0167		-619.04	1250.08	1290.55	6
M6	0.406	0.418	∞	0.00083	1.429				0.0167		-619.04	1248.08	1281.80	5
M7	0.406	0.418	∞	0.00083	1.428			∞	0.0167		-619.04	1250.08	1290.55	6
M8	0.419	0.397	∞	0.00057	1.428			20	0.0222		-608.46	1228.91	1269.39	6
M9	0.419	0.397	∞	0.00057	1.428			20	0.0222		-608.46	1230.91	1278.13	7
M10	0.333	0.287	∞	0.00036	9344.48	0.348	0.478	∞	0.0349	1.458	-600.64	1221.27	1288.73	10
M11	0.332	0.286	∞	0.00035	6517.98	0.348	0.478	∞	0.0349	1.458	-600.60	1219.20	1279.91	9
M12	0.406	0.418	∞	0.00082	0				0.0166	1.420	-619.10	1250.20	1290.68	6
M13	0.407	0.418	∞	0.00083	0				0.0167	1.421	-619.10	1248.20	1281.93	5
M14	0.407	0.418	∞	0.00083	0			∞	0.0167	1.420	-619.10	1250.20	1290.68	6
M15	0.419	0.397	∞	0.00057	0			∞	0.0222	1.420	-608.52	1229.04	1269.52	6
M16	0.419	0.397	∞	0.00057	0			∞	0.0222	1.420	-608.52	1231.04	1278.26	7
M17	0.332	0.286	∞	0.00035	0	0.348	0.477	∞	0.0349	1.458	-600.76	1219.52	1280.23	9
M18	0.332	0.286	∞	0.00035	0	0.348	0.478	∞	0.0349	1.458	-600.76	1217.52	1271.48	8
M19	0.405	0.410	∞	0.00420	1.437	0.372					-647.94	1307.88	1348.35	6
M20	0.381	0.343	∞	0.00043	1.430	0.230			0.0272		-601.89	1217.79	1265.01	7
M21	0.405	0.410	∞	0.00420	1.437	0.372					-647.94	1305.88	1339.61	5
M22	0.381	0.343	∞	0.00044	1.430	0.230			0.0272		-601.89	1215.79	1256.26	6
M23	0.406	0.415	∞	0.00427	1.439		0.427				-648.71	1309.43	1349.90	6
M24	0.335	0.289	∞	0.00036	1.464		0.464		0.0343		-600.68	1215.36	1262.58	7
M25	0.406	0.416	∞	0.00427	1.439		0.427				-648.71	1307.43	1341.15	5
M26*	0.334	0.288	∞	0.00036	1.465		0.464		0.0343		-600.68	1213.36	1253.83	6
M27	0.502	0.507	∞	0.00392	1.309	0.117	0.146				-638.64	1291.28	1338.50	7
M28	0.332	0.285	∞	0.00035	1.467	0.348	0.478		0.0349		-600.67	1217.34	1271.31	8
M29	0.502	0.507	∞	0.00391	1.309	0.117	0.146				-638.64	1289.28	1329.75	6
M30	0.332	0.285	∞	0.00035	1.467	0.348	0.478		0.0349		-600.67	1215.34	1262.56	7

Model	λ^c	μ	K	γ before shift	λ^a	γ after shift	Time shift (Ma)	Loglik	AIC	BIC	Npars
M31	0.33	0.30	∞	0.0007	1.469	0.008	5.59	-635.81	1283.62	1324.09	6
M32 (bats)	0.34	0.49	∞	0.0048	1.40	0.036	52.15	-599.07	1222.14	1303.09	12
M32 non volant	0.34	0.30	∞	0.006	0.10	1E-13	14.69				

Table S4 – ML parameters based on fitting the M26 model to the maximum clade credibility tree and posterior distribution of trees, for the main dataset (D1). Obtained by fitting DAISIE to the data from the maximum clade credibility tree, and to 1000 trees of the posterior. Results for the analysis of the main scenario (D1, DNA-only trees, 88 million years island age, mainland pool size 1000 species, high human impact, CS1). Rates in events per lineage per million years.

**M26 model**	**λ^c Cladogenesis (non-volant and bats)**	**μ Extinction (non-volant)**	**γ Colonization (non-volant)**	**λ^a Anagenesis (non-volant and bats)**	**μ Extinction (bats)**	**γ Colonization (bats)**
MCC tree	0.334	0.288	0.00036	1.465	0.464	0.034
Posterior mean	0.312	0.262	0.00033	1.569	0.450	0.035
Percentile 0.025	0.269	0.220	0.00027	1.176	0.395	0.030
Percentile 0.975	0.357	0.309	0.00038	2.123	0.502	0.038

Table S5 – **Malagasy mammal diversity at different stages**. Species diversity counts of Malagasy mammals are shown for the two scenarios of past human influence, for the current state and for potential future scenarios in which all species threatened in 2010, 2015 and 2021, respectively, go extinct.

	**Pre-human diversity (Low human impact)**	**Pre-human diversity (High human impact)**	**Current diversity**
**All species**	235	249	219
**Bats**	44	46	44
**Non-volant**	191	203	175

	**Diversity if threatened go extinct (IUCN 2010)**	**Diversity if threatened go extinct (IUCN 2015)**	**Diversity if threatened go extinct (IUCN 2021)**
**All species**	163	109	91
**Bats**	41	40	39
**Non-volant**	122	69	52

Table S6 - Sensitivity analysis of ERTs. ERT values (Myr) are shown per dataset and per diversity to be regained (return to pre-human diversity or return to contemporary diversity if threatened species from IUCN 2010, 2015 and 2021 go extinct). Based on the mean node values from the maximum clade credibility tree for each dataset. Results shown for the preferred model (M26; best model overall, different rates of extinction and colonization for bats). Sampling column indicates taxon sampling per dataset in the phylogenies by Upham et al.[45]. M column shows mainland pool size used per dataset. Island age column shows the age of Madagascar (in Myr). Colonization scenario column shows that used per dataset as presented in Table S1. Human impact column shows scenario of past human impact used per dataset. Numbers in parentheses indicate ERT to regain 95% of the target diversity.

						Return to pre-human		IUCN 2010 threatened extinct to current diversity		IUCN 2015 threatened extinct to current diversity		IUCN 2021 threatened extinct to current diversity	
**Dataset**	**Sampling**	**M**	**Island age (Myr)**	**Colonization scenario**	**Human impact**	**Non-volant mammals**	**Bats**	**Non-volant mammals**	**Bats**	**Non-volant mammals**	**Bats**	**Non-volant mammals**	**Bats**
D1	DNA-only	1000	88	CS1	High	3.13 (2.05)	1.3 (0)	7.54 (6.46)	1.61 (0.41)	19.16 (18.08)	2.06 (0.88)	24.75 (23.67)	2.49 (1.31)
D2	DNA-only	2000	88	CS1	High	3.13 (2.05)	1.30 (0)	7.55 (6.47)	1.61 (0.41)	19.20 (18.12)	2.07 (0.88)	24.80 (23.72)	2.51 (1.31)
D3	DNA-only	5000	88	CS1	High	3.14 (2.05)	1.30 (0)	7.57 (6.49)	1.61 (0.40)	19.24 (18.16)	2.08 (0.87)	24.85 (23.77)	2.51 (1.31)
D4	DNA-only	1000	66	CS1	High	2.96 (1.93)	1.32 (0)	7.12 (6.10)	1.63 (0.42)	18.08 (17.06)	2.09 (0.89)	23.33 (22.31)	2.52 (1.33)
D5	DNA-only	2000	66	CS1	High	2.96 (1.94)	1.32 (0)	7.13 (6.11)	1.63 (0.41)	18.10 (17.08)	2.10 (0.89)	23.36 (22.34)	2.54 (1.33)
D6	DNA-only	5000	66	CS1	High	2.97 (1.94)	1.32 (0)	7.14 (6.12)	1.63 (0.41)	18.14 (17.11)	2.11 (0.88)	23.40 (22.38)	2.55 (1.33)
D7	DNA-only	1000	88	CS1	Low	1.85 (0.76)	0 (0)	7.56 (6.48)	1.83 (0.47)	19.22 (18.14)	2.34 (0.99)	24.84 (23.76)	2.82 (1.47)
D8	DNA-only	1000	88	CS2	High	3.90 (2.55)	2.22 (0)	9.31 (7.97)	2.42 (0.60)	23.31 (21.97)	3.06 (1.26)	29.84 (28.50)	3.65 (1.85)
D9	DNA-only	1000	88	CS2	Low	1.79 (0.74)	0 (0)	7.32 (6.28)	1.41 (0.36)	18.65 (17.61)	1.81 (0.77)	24.12 (23.08)	2.19 (1.15)
D10	Completed	1000	88	CS1	High	3.25 (2.12)	1.73 (0)	7.82 (6.70)	1.92 (0.48)	19.85 (18.74)	2.43 (1.00)	25.63 (24.51)	2.90 (1.47)
D11	Completed	1000	88	CS1	Low	1.91 (0.79)	0 (0)	7.82 (6.70)	2.32 (0.57)	19.88 (18.76)	2.91 (1.17)	25.67 (24.55)	3.44 (1.71)
D12	Completed	1000	88	CS2	High	3.43 (2.24)	0.92 (0)	8.22 (7.04)	1.12 (0.29)	20.80 (19.62)	1.44 (0.61)	26.79 (25.61)	1.74 (0.91)
D13	Completed	1000	88	CS2	Low	1.90 (0.78)	0 (0)	7.75 (6.64)	1.32 (0.33)	19.70 (18.59)	1.68 (0.71)	25.43 (24.32)	2.01 (1.04)

Table S7 – Evolutionary return times (ERTs) estimated based on the posterior distribution of trees of the main dataset (D1). Based on 1000 trees of the posterior. Mean ERT values are given per diversity target for non-volant mammals and bats. Values in brackets indicate the 2.5 – 97.5 percentiles. Values in italics indicate the ERTs to regain 95% of the target diversity. ERTs in Myr.

	**Current diversity to pre-human diversity**	**IUCN 2010 threatened extinct to current diversity**	**IUCN 2015 threatened extinct to current diversity**	**IUCN 2021 threatened extinct to current diversity**
**Non-volant**	2.93 (2.30 - 3.60)<br>*1.92 (1.51 - 2.35)*	7.07 (5.57 - 8.65)<br>*6.06 (4.77 - 7.42)*	18.01 (14.24 - 21.9)<br>*17.00 (13.44 - 20.72)*	23.29 (18.45 - 28.3)<br>*22.28 (17.66 - 27.06)*
**Bats**	1.62 (1.20 - 2.20)<br>*0 (0 - 0)*	1.90 (1.49 - 2.39)<br>*0.48 (0.38 - 0.59)*	2.42 (1.92 - 3.02)<br>*1.02 (0.82 - 1.24)*	2.91 (2.32 - 3.6)<br>*1.51 (1.23 - 1.83)*

Table S8 – Evolutionary return times (ERTs) under different scenarios of uncertainty, main dataset D1. Scenario A - Pessimistic scenario where all currently non-evaluated and all currently threatened species (IUCN 2021) go extinct; Scenario B - only currently Critically Endangered (CR) species (IUCN 2021) go extinct; Scenario C - currently threatened species (IUCN 2021) go extinct, but excluding those species that have changed from a non-evaluated to a threat category between 2010 and 2021, and those species that have changed from a non-threat to a threat category between 2010 and 2021 due to taxonomic changes. Based on 1000 trees of the posterior. Mean ERT values are given per diversity target for non-volant mammals and bats. Values in brackets indicate the 2.5 – 97.5 percentiles. ERTs in Myr.

	**ERT Scenario A**	**ERT Scenario B**	**ERT Scenario C**
Bats	6.6 (5.5 - 7.8)	0 (no CR species)	2.42 (1.92 - 3.02)
Non-volant mammals	26.2 (20.8 - 32)	4.1 (3.23 - 5.03)	8.56 (6.74 - 10.47)

Table S9 – Evolutionary return times (ERTs) for recovering the number of endemic species on Madagascar. This is equivalent to recovering global diversity. Estimated based on the posterior distribution of trees of the main dataset (D1). Based on 1000 trees of the posterior. Mean ERT values are given per diversity target for non-volant mammals and bats. Values in brackets indicate the 2.5 – 97.5 percentiles. ERTs in Myr. For non-volant mammals ERTs for endemic species are slightly higher than for total species (Table S7); for bats ERTs for endemic species are lower than for total species (Table S7).

	**Current diversity to pre-human endemic diversity**	**IUCN 2010 threatened extinct to current endemic diversity**	**IUCN 2015 threatened extinct to current endemic diversity**	**IUCN 2021 threatened extinct to current diversity**
**Non-volant**	2.94 (2.32-3.61)	7.09 (5.6-8.67)	18.03 (14.3-21.98)	23.32 (18.53-28.33)
**Bats**	0.22 (0.18-0.26)	0.31 (0.26-0.37)	0.41 (0.35-0.48)	0.53 (0.43-0.69)

Supplementary References

81. Benoit, J. *et al.* Comparative anatomy and three-dimensional geometric-morphometric study of the bony labyrinth of Bibymalagasia (Mammalia, Afrotheria). *Journal of Vertebrate Paleontology* **35**, e930043 (2015).
82. Buckley, M. A Molecular Phylogeny of *Plesiorycteropus* Reassigns the Extinct Mammalian Order 'Bibymalagasia'. *PLOS ONE* **8**, e59614 (2013).
83. Demos, T. C. *et al.* Multilocus phylogeny of a cryptic radiation of Afrotropical long-fingered bats (Chiroptera, Miniopteridae). *Zoologica Scripta* **49**, 1–13 (2020).
