# Supplementary material for: The macroevolutionary impact of recent and imminent mammal extinctions on Madagascar
Source: Nat Commun. 2023 Jan 10;14:14. doi: 10.1038/s41467-022-35215-3 (PMC9832013; doi:10.1038/s41467-022-35215-3)
Supplement: Supplementary file 3 — Description of Additional Supplementary Files [file 41467_2022_35215_MOESM3_ESM.docx]

**Supplementary Data 1**

File Name: Data S1_Mammal species Madagascar

Description: Checklist of non-marine mammal species present on Madagascar before humans arrived, including information on 2010, 2015, and 2021 IUCN threat status, taxonomy notes, colonization scenario, endemicity, additional range notes, and references used in the collection of the data.

**Supplementary Data 2**

File Name: Data S2_Colonization scenario1

Description: Colonization time across the posterior distribution of trees (1000 trees) for colonization scenario 1.

**Supplementary Data 3**

File Name: Data S3_Colonization scenario2

Description: Colonization time across the posterior distribution of trees (1000 trees) for colonization scenario 2.

**Supplementary Data 4**

File Name: Data S4_Sensitivity analyses

Description: Maximum-likelihood parameters of the best overall model for each of 13 scenarios using BIC or AIC. DAISIE was fitted to the maximum clade credibility tree for each scenario.

**Supplementary Data 5**

File Name: Data S5_IUCN Uplistings

Description: List of Malagasy mammal species that have been uplisted between 2010 and 2021, including whether uplisting was due to legitimate threat increase or taxonomy influenced.

**Supplementary Data 6**

File Name: References_Data_S1_and_S5

References cited in Supplementary Data 1 and 5.
