# Supplementary material for: The macroevolutionary impact of recent and imminent mammal extinctions on Madagascar
Source: Nat Commun. 2023 Jan 10;14:14. doi: 10.1038/s41467-022-35215-3 (PMC9832013; doi:10.1038/s41467-022-35215-3)
Supplement: Supplementary file 9 — Supplementary Data 6 [file 41467_2022_35215_MOESM9_ESM.docx]

**References used to compile the checklist of Malagasy mammals (Data S1), the extinction data (Data S1) and the taxonomic state changes for recently up-listed species in the IUCN Red List (Data S5)**

1. Veron, G. *et al.* New insights into the systematics of Malagasy mongoose‐like carnivorans (Carnivora, Eupleridae, Galidiinae) based on mitochondrial and nuclear DNA sequences. *J. Zool. Syst. Evol. Res.* **55**, 250–264 (2017).

2. Mittermeier, R. A. *et al.* Lemur diversity in Madagascar. *Int. J. Primatol.* **29**, 1607–1656 (2008).

3. Kappeler, P. Morphology, behaviour and molecular evolution of giant mouse lemurs (*Mirza* spp.) Gray, 1870, with description of a new species. *Primate Rep* **71**, 3–26 (2005).

4. Wilson, D. E. & Reeder, D. M. *Mammal species of the world: a taxonomic and geographic reference*. vol. 1 (JHU Press, 2005).

5. Goodman, S. M. & Helgen, K. M. Species limits and distribution of the Malagasy carnivoran genus *Eupleres* (Family Eupleridae). *Mammalia* **74**, 177–185 (2010).

6. Andriaholinirina, N. *et al.* Molecular phylogeny and taxonomic revision of the sportive lemurs (*Lepilemur*, Primates). *BMC Evol. Biol.* **6**, 17 (2006).

7. Frasier, C. *et al.* A New Species of Dwarf Lemur (Cheirogaleidae: *Cheirogaleus medius* Group) from the Ankarana and Andrafiamena-Andavakoera Massifs, Madagascar. *Primate Conserv.* **30**, 59–72 (2016).

8. Groeneveld, L. F., Weisrock, D. W., Rasoloarison, R. M., Yoder, A. D. & Kappeler, P. M. Species delimitation in lemurs: Multiple genetic loci reveal low levels of species diversity in the genus Cheirogaleus. *BMC Evol. Biol.* **9**, 1–16 (2009).

9. Hotaling, S. *et al.* Species discovery and validation in a cryptic radiation of endangered primates: Coalescent-based species delimitation in Madagascar’s mouse lemurs. *Mol. Ecol.* **25**, 2029–2045 (2016).

10. International Union for Conservation of Nature. *IUCN Red List of Threatened Species. Version 2021-2.* https://www.iucnredlist.org/en (2021).

11. International Union for Conservation of Nature. *IUCN Red List of Threatened Species. Version 2015-3.* https://www.iucnredlist.org/en (2015).

12. International Union for Conservation of Nature. *IUCN Red List of Threatened Species. Version 2010.4.* https://www.iucnredlist.org/en (2010).

13. Kouvari, M. & van der Geer, A. A. E. Biogeography of extinction: The demise of insular mammals from the Late Pleistocene till today. *Palaeogeogr. Palaeoclimatol. Palaeoecol.* **505**, 295–304 (2018).

14. Goodman, S. M., Zafindranoro, H. H., & Soarimalala, V. A case of the sympatric occurrence of *Microgale brevicaudata* and *M. grandidieri* (Afrosoricida, Tenrecidae) in the Beanka Forest, Maintirano. *Malagassy Nature* **5**, 104-108 (2011).

15. Everson, K. M., Soarimalala, V., Goodman, S. M. & Olson, L. E. Multiple loci and complete taxonomic sampling resolve the phylogeny and biogeographic history of tenrecs (Mammalia: Tenrecidae) and reveal higher speciation rates in Madagascar’s humid forests. *Syst. Biol.* **65**, 890–909 (2016).

16. Goodman, S. M. & Jungers, W. L. *Extinct Madagascar: Picturing the island’s past*. (University of Chicago Press, 2014).

17. Soarimalala, V., Raheriarisena, M. & Goodman, S. M. New distributional records from central-eastern Madagascar and patterns of morphological variation in the endangered shrew tenrec *Microgale jobihely* (Afrosoricida: Tenrecidae). *Mammalia* **74**, 187–198 (2010).

18. Jenkins, P. D. & Goodman, S. M. A new species of *Microgale* (Lipotyphla, Tenrecidae) from isolated forest in southwestern Madagascar. *Bull. Nat. Hist. Mus. Zool. Ser.* **65**, 155–164 (1999).

19. Olson, L. E. *et al.* Phylogeography of *Microgale brevicaudata* (Tenrecidae) and Description of a New Species from Western Madagascar. *J. Mammal.* **90**, 1095–1110 (2009).

20. Jenkins, P. D. Description of a new species of *Microgale* (Insectivora: Tenrecidae) from eastern Madagascar. *Bull. Br. Mus. Nat. Hist., Zool* **58**(1), 53–59 (1992).

21. Jenkins, P. D., Raxworthy, C. J. & Nussbaum, R. A. A new species of Microgale (Insectivora, Tenrecidae), with comments on the status of four other taxa of shrew tenrecs. *Bull. Nat. Hist. Mus. Zool. Ser.* **63**, 1–12 (1997).

22. Jenkins, P. D., Goodman, S. M., & Raxworthy, C. J. The shrew tenrecs (Microgale) (Insectivora: Tenrecidae) of the Reserve Naturelle Integrale d’Andringitra, Madagascar. *Fieldiana Zoology. A Floral and faunal inventory of the eastern slopes of the Réserve naturelle intégrale d’Andringitra, Madagascar: With reference to elevational variation*, **85**, 191–217 (1996).

23. Goodman, S. M., & Jenkins, P. D. The insectivores of the Réserve Spéciale d’Anjanaharibe-Sud, Madagascar. *Fieldiana Zoology.* *A floral and faunal inventory of the Réserve spéciale d’Anjanaharibe-Sud, Madagascar: With reference to elevational variation*, **90**, 139–161 (1998).

24. Jenkins, P. D. A new species of *Microgale* (Insectivora, Tenrecidae) from eastern Madagascar with an unusual dentition. *American Museum novitates*, **3067** (1993).

25. Goodman, S., & Soarimalala, V. A new species of *Microgale* (Lipotyphla: Tenrecidae: Oryzorictinae) from the Forêt des Mikea of southwestern Madagascar. *Proceedings of the Biological Society of Washington* **117**(3):25, 1-265. (2004).

26. Goodman, S., Raxworthy, C., Maminirina, C. & Olson, L. A new species of shrew tenrec (*Microgale jobihely*) from northern Madagascar. *J. Zool.* **270**, 384–398 (2006).

27. Werdelin, L. Bibymalagasia (Mammalia Incertae sedis). in *Cenozoic Mammals of Africa* (eds. Werdelin, L., Sanders, W.J.) 113–114 (University of California Press, 2010).

28. Buckley, M. A molecular phylogeny of Plesiorycteropus reassigns the extinct mammalian order ‘Bibymalagasia’. *PLOS ONE* **8**, e59614 (2013).

29. Faure, M., Guérin, C., Genty, D., Gommery, D. & Ramanivosoa, B. The oldest fossil hippopotamus (*Hippopotamus laloumena*) of Madagascar (Belobaka, Mahajanga Province). *Comptes Rendus Palevol* **9**, 155–162 (2010).

30. Faure, M., & Guerin, C. *Hippopotamus laloumena* nov. Sp., la troisième espèce d’hippopotame holocène de Madagascar. *Proceedings of the Academy of Sciences. Series II. Mechanics. Physics. Chemistry. Space sciences. Earth sciences.*, **310**(9), 1299–1305 (1990).

31. Rosenberger, A. L. *et al.* Giant subfossil lemur graveyard discovered, submerged, in Madagascar. *J. Hum. Evol.* **81**, 83–87 (2015).

32. Rakotovao, M., Lignereux, Y., Orliac, M. J., Duranthon, F. & Antoine, P.-O. *Hippopotamus lemerlei* Grandidier, 1868 et *Hippopotamus madagascariensis* Guldberg, 1883 (Mammalia, Hippopotamidae): Anatomie crânio-dentaire et révision systématique. *Geodiversitas* **36**, 117–161 (2014).

33. Goodman, S. M. *Les Carnivora de Madagascar*. (Association Vahatra, 2012).

34. Gerber, B. D., Karpanty, S. M. & Randrianantenaina, J. Activity patterns of carnivores in the rain forests of Madagascar: Implications for species coexistence. *J. Mammal.* **93**, 667–676 (2012).

35. Burney, D. A. *et al.* Subfossil lemur discoveries from the Beanka Protected Area in western Madagascar. *Quat. Res. U. S.* **93**, 187–203 (2020).

36. Goodman, S. M. *Les Chauves-Souris de Madagascar*. (Association Vahatra, 2012).

37. Naidoo, T., Goodman, S. M., Schoeman, M. C., Taylor, P. J. & Lamb, J. M. Partial support for the classical ring species hypothesis in the *Chaerephon pumilus* species complex (Chiroptera: Molossidae) from southeastern Africa and western Indian Ocean islands. *Mammalia* **80**, 627–643 (2016).

38. Goodman, S. M., Buccas, W., Naidoo, T., Ratrimomanarivo, F., Taylor, P. J., & Lamb, J. Patterns of morphological and genetic variation in western Indian Ocean members of the *Chaerephon* ‘*pumilus*’ complex (Chiroptera: Molossidae), with the description of a new species from Madagascar. *Zootaxa*, **2551**(1), 1–36 (2010). https://doi.org/10.11646/zootaxa.2551.1.1

39. Cardiff, S. G. & Jenkins, R. K. The bats of Madagascar: A conservation challenge. *Lessons Conserv.* **6**, 80–108 (2016).

40. Goodman, S., Weyeneth, N., Ibrahim, Y., Saïd, I. & Ruedi, M. A review of the bat fauna of the Comoro Archipelago. *Acta Chiropterologica* **12**, 117–141 (2010).

41. Goodman, S. M. *et al.* An integrative approach to characterize Malagasy bats of the subfamily Vespertilioninae Gray, 1821, with the description of a new species of *Hypsugo*. *Zool. J. Linn. Soc.* **173**, 988–1018 (2015).

42. Monadjem, A. *et al.* A revision of pipistrelle-like bats (Mammalia: Chiroptera: Vespertilionidae) in East Africa with the description of new genera and species. *Zool. J. Linn. Soc.* **191**, 1114–1146 (2020).

43. Alumbaugh, J. L., Goodman, S. M. & Samonds, K. E. Morphometric analyses of modern and subfossil *Macronycteris* (family Hipposideridae) refine groups from Anjohibe Cave, northwestern Madagascar. *Acta Chiropterologica* **23**, 33–43 (2021).

44. Goodman, S. M., Schoeman, M. C., Rakotoarivelo, A. & Willows-Munro, S. How many species of *Hipposideros* have occurred on Madagascar since the Late Pleistocene? *Zool. J. Linn. Soc.* **177**, 428–449 (2016).

45. Goodman, S. M. *et al.* The use of molecular and morphological characters to resolve the taxonomic identity of cryptic species: The case of *Miniopterus manavi* (Chiroptera, Miniopteridae). *Zool. Scr.* **38**, 339–363 (2009).

46. Reher, S., Rabarison, H., Schoroth, M. & Dausmann, K. Seasonal movements of insectivorous bat species in southwestern Madagascar. *Malagasy Nat.* **13**, 117–124 (2019).

47. Foley, N. M., Goodman, S. M., Whelan, C. V., Puechmaille, S. J. & Teeling, E. Towards navigating the Minotaur’s labyrinth: Cryptic diversity and taxonomic revision within the speciose genus *Hipposideros* (Hipposideridae). *Acta Chiropterologica* **19**, 1–18 (2017).

48. Christidis, L., Goodman, S. M., Naughton, K. & Appleton, B. Insights into the evolution of a cryptic radiation of bats: Dispersal and ecological radiation of Malagasy *Miniopterus* (Chiroptera: Miniopteridae). *PLOS ONE* **9**, e92440 (2014).

49. Goodman, S. M., Ramasindrazana, B., Maminirina, C. P., Schoeman, M. C., & Appleton, B. Morphological, bioacoustical, and genetic variation in *Miniopterus* bats from eastern Madagascar, with the description of a new species. *Zootaxa*, **2880**(1), 1–19 (2011). https://doi.org/10.11646/zootaxa.2880.1.1

50. Goodman, S. M., Maminirina, C. P., Bradman, H. M., Christidis, L. & Appleton, B. R. Patterns of morphological and genetic variation in the endemic Malagasy bat *Miniopterus gleni* (Chiroptera: Miniopteridae), with the description of a new species, *M. griffithsi*. *J. Zool. Syst. Evol. Res.* **48**, 75–86 (2010).

51. Goodman, S. M. & Maminirina, C. P. Specimen records referred to *Miniopterus majori* Thomas, 1906 (Chiroptera) from the Comoros Islands. *Mammalia* **71**, 151–156 (2007).

52. Goodman, S. M. *et al.* A new species of *Miniopterus* (Chiroptera: Miniopteridae) from lowland southeastern Madagascar. *Mamm. Biol.* **73**, 199–213 (2008).

53. Goodman, S. M. *et al.* Specific status of populations on Madagascar referred to *Miniopterus fraterculus* (Chiroptera: Vespertilionidae), with description of a new species. *J. Mammal.* **88**, 1216–1229 (2007).

54. Goodman, S. M. & Ramasindrazana, B. Description of a new species of the *Miniopterus aelleni* group (Chiroptera: Miniopteridae) from upland areas of central and northern Madagascar. *Zootaxa* **3936**, 538 (2015).

55. Upham, N. S., Esselstyn, J. A. & Jetz, W. Inferring the mammal tree: Species-level sets of phylogenies for questions in ecology, evolution, and conservation. *PLOS Biol.* **17**, e3000494 (2019).

56. Dammhahn, M., Rakotondramanana, C. F. & Goodman, S. M. Coexistence of morphologically similar bats (Vespertilionidae) on Madagascar: Stable isotopes reveal fine-grained niche differentiation among cryptic species. *J. Trop. Ecol.* **31**, 153–164 (2015).

57. Goodman, S. M., Rakotondraparany, F. & Kofoky, A. The description of a new species of *Myzopoda* (Myzopodidae: Chiroptera) from western Madagascar. *Mamm. Biol.* **72**, 65–81 (2007).

58. Lamb, J. M. *et al.* Phylogeography and predicted distribution of African-Arabian and Malagasy populations of giant mastiff bats, *Otomops* spp. (Chiroptera: Molossidae). *Acta Chiropterologica* **10**, 21–40 (2008).

59. Benda, P. & Vallo, P. Taxonomic revision of the genus *Triaenops* (Chiroptera: Hipposideridae) with description of a new species from Southern Arabia and definitions of a new genus and tribe. **58**, (2009).

60. Russell, A. L., Ranivo, J., Palkovacs, E. P., Goodman, S. M. & Yoder, A. D. Working at the interface of phylogenetics and population genetics: A biogeographical analysis of *Triaenops* spp. (Chiroptera: Hipposideridae). *Mol. Ecol.* **16**, 839–851 (2007).

61. Werdelin, L. & Sanders, W. J. *Cenozoic Mammals of Africa*. (Univ of California Press, 2010).

62. Goodman, S. M. & Jenkins, R. K. B. A review of the genus *Scotophilus* (Mammalia, Chiroptera, Vespertilionidae) on Madagascar, with the description of a new species. *Zoosystema* **27**, 867–882 (2005).

63. Goodman, S. M. & Ranivo, J. The geographical origin of the type specimens of *Triaenops rufus* and *T. humbloti* (Chiroptera: Hipposideridae) reputed to be from Madagascar and the description of a replacement species name. *Mammalia* **73**, 47–55 (2009).

64. Mittermeier, R. A. *et al.* *Lémuriens de Madagascar*. (Muséum national d’Histoire naturelle, 2014).

65. Groves, C. The taxonomy of Cheirogaleidae: An ever-expanding species list. in *The Dwarf and Mouse Lemurs of Madagascar* (eds. Lehman, S. M., Radespiel, U. & Zimmermann, E.) 21–53 (Cambridge University Press, 2016). doi:10.1017/CBO9781139871822.003.

66. Herrera, J. P. & Dávalos, L. M. Phylogeny and divergence times of lemurs inferred with recent and ancient fossils in the tree. *Syst. Biol.* **65**, 772–91 (2016).

67. Andriantompohavana, R. *Molecular phylogeny and taxonomic revision of the woolly lemurs, genus* Avahi *(primates: lemuriformes)*. vol. 51 (Museum of Texas Tech University, 2007).

68. Herrera, J. P. Testing the adaptive radiation hypothesis for the lemurs of Madagascar. *R. Soc. Open Sci.* **4**, 161014 (2017).

69. Zaramody, A. Molecular phylogeny and taxonomic revision of the eastern woolly lemurs (*Avahi laniger*). *Prim Rep* **74**, 9–22 (2006).

70. Lei, R. Nocturnal lemur diversity at Masoala National Park. *Mus. Tex. Tech Univ. Spec. Publ.* **53**, 1–41 (2008).

71. Sawyer, R. M., Fenosoa, Z. S. E., Andrianarimisa, A. & Donati, G. The effect of habitat disturbance on the abundance of nocturnal lemur species on the Masoala Peninsula, northeastern Madagascar. *Primates* **58**, 187–197 (2017).

72. Fenosoa, Z. *et al.* Height and support use of three sympatric nocturnal lemurs in different habitats varying in disturbance on the Masoala Peninsula. *Lemur News* **21**, 39–43 (2018).

73. Lei, R. *et al.* A new species in the genus *Cheirogaleus* (Cheirogaleidae). *Primate Conserv.* **29**, 43–54 (2015).

74. Lei, R. *et al.* Revision of Madagascar’s dwarf lemurs (Cheirogaleidae: *Cheirogaleus*): Designation of species, candidate species status and geographic boundaries based on molecular and morphological data. *Primate Conserv.* **28**, 9–35 (2014).

75. Thiele, D., Razafimahatratra, E. & Hapke, A. Discrepant partitioning of genetic diversity in mouse lemurs and dwarf lemurs – Biological reality or taxonomic bias? *Mol. Phylogenet. Evol.* **69**, 593–609 (2013).

76. Groeneveld, L. F., Rasoloarison, R. M., & Kappeler, P. M. Morphometrics confirm taxonomic deflation in dwarf lemurs (Primates: Cheirogaleidae), as suggested by genetics. *Zoological Journal of the Linnean Society*, **161**(1), 229–244 (2011). https://doi.org/10.1111/j.1096-3642.2010.00634.x

77. McLain, A. T. *et al.* A new *Cheirogaleus* (Cheirogaleidae: *Cheirogaleus crossleyi* group) species from southeastern Madagascar. *Primate Conserv.* **31**, 27–36 (2017).

78. Sato, H. *et al.* Dietary flexibility and feeding strategies of *Eulemur*: A comparison with *Propithecus*. *Int. J. Primatol.* **37**, 109–129 (2016).

79. Rakotonirina, L. H. F. *et al.* A preliminary assessment of Sifaka (*Propithecus*) distribution, chromatic variation and conservation in western central Madagascar. *Primate Conserv.* **28**, 43–53 (2014).

80. Kistler, L. *et al.* Comparative and population mitogenomic analyses of Madagascar’s extinct, giant ‘subfossil’ lemurs. *J. Hum. Evol.* **79**, 45–54 (2015).

81. Lei, R. *et al.* Phylogenomic reconstruction of sportive lemurs (genus *Lepilemur*) recovered from mitogenomes with inferences for Madagascar biogeography. *J. Hered.* **108**, 107–119 (2017).

82. Louis, E. J. Molecular and morphological analyses of the sportive lemurs (Family: Megaladapidae, Genus: *Lepilemur*) reveals 11 previously unrecognized species. *Mus. Tex. Tech Univ. Spec. Publ.* **49**, 1–47 (2006).

83. Ramaromilanto, B. *et al.* Sportive lemur diversity at Mananara-Nord Biosphere Reserve, Madagascar. *Occas. Pap. Mus. Tex. Tech Univ.* **286**, 1–22 (2009).

84. Louis, E. E. *et al.* Revision of the mouse lemurs, *Microcebus* (Primates, Lemuriformes), of northern and northwestern Madagascar with descriptions of two new species at Montagne d’Ambre National Park and Antafondro Classified Forest. *Primate Conserv.* **23**, 19–38 (2008).

85. Sgarlata, G. M. *et al.* Genetic and morphological diversity of mouse lemurs (*Microcebus* spp.) in northern Madagascar: The discovery of a putative new species? *Am. J. Primatol.* **81**, e23070 (2019).

86. Olivieri, G. *et al.* The ever-increasing diversity in mouse lemurs: Three new species in north and northwestern Madagascar. *Mol. Phylogenet. Evol.* **43**, 309–327 (2007).

87. Setash, C. M., Zohdy, S., Gerber, B. D. & Karanewsky, C. J. A biogeographical perspective on the variation in mouse lemur density throughout Madagascar. *Mammal Rev.* **47**, 212–229 (2017).

88. Radespiel, U. *et al.* First indications of a highland specialist among mouse lemurs (*Microcebus* spp.) and evidence for a new mouse lemur species from eastern Madagascar. *Primates* **53**, 157–170 (2012).

89. Radespiel, U. *et al.* Exceptional diversity of mouse lemurs (*Microcebus* spp.) in the Makira region with the description of one new species. *Am. J. Primatol.* **70**, 1033–1046 (2008).

90. Andriantompohavana, R. Mouse lemurs of northwestern Madagascar with a description of a new species at Lokobe Special Reserve. *Occas Pap Tex Tech Univ Mus* **259**, 1–23 (2006).

91. Rasoloarison, R. M., Weisrock, D. W., Yoder, A. D., Rakotondravony, D. & Kappeler, P. M. Two new species of mouse lemurs (Cheirogaleidae: *Microcebus*) from eastern Madagascar. *Int. J. Primatol.* **34**, 455–469 (2013).

92. Rasolooarison, R. M., Goodman, S. M. & Ganzhorn, J. U. Taxonomic revision of mouse Lemurs (*Microcebus*) in the western portions of Madagascar. *Int. J. Primatol.* **21**, 963–1019 (2000).

93. Roos, C. & Kappeler, P. Distribution and conservation status of two newly described Cheirogaleid species, *Mirza zaza* and *Microcebus lehilahytsara*. *Primate Conserv.* **21**, 51–53 (2006).

94. Zimmermann, E., Cepok, S., Rakotoarison, N., Zietemann, V. & Radespiel, U. Sympatric mouse lemurs in North-West Madagascar: A new rufous mouse lemur species (*Microcebus ravelobensis*). *Folia Primatol. (Basel)* **69**, 106–114 (1998).

95. Schüßler, D. *et al.* Ecology and morphology of mouse lemurs (*Microcebus* spp.) in a hotspot of microendemism in northeastern Madagascar, with the description of a new species. *Am. J. Primatol.* **82**, e23180 (2020).

96. Gommery, D., Ramanivosoa, B., Tombomiadana-Raveloson, S., Randrianantenaina, H. & Kerloc’h, P. Une nouvelle espèce de lémurien géant subfossile du Nord-Ouest de Madagascar (*Palaeopropithecus kelyus*, Primates). *Comptes Rendus Palevol* **8**, 471–480 (2009).

97. Mein, P. *et al.* Nouvelles espèces subfossiles de rongeurs du Nord-Ouest de Madagascar. *Comptes Rendus Palevol* **9**, 101–112 (2010).

98. Amori, G., Gippoliti, S. & Helgen, K. M. Diversity, distribution, and conservation of endemic island rodents. *Quat. Int.* **182**, 6–15 (2008).

99. Ding, X. *et al.* Sperm morphology in the Malagasy rodents (Muroidea: Nesomyinae). *J. Morphol.* **271**, 1493–1500 (2010).

100. Jansa, S. A., Carleton, M. D., Soarimalala, V., Rakotomalala, Z. & Goodman, S. M. A Review of the *Eliurus tanala* complex (Rodentia: Muroidea: Nesomyidae), with description of a new species from dry forests of western Madagascar. *Bull. Am. Mus. Nat. Hist.* **2019**, 1–69 (2019).

101. Carleton, M. D., Goodman, S. M. & Rakotondravony, D. A new species of tufted-tailed rat, genus *Eliurus* (Muridae: Nesomyinae), from western Madagascar, with notes on the distribution of *E. myoxinus*. *Proc. Biol. Soc. Wash.* **114**(4), 972–987 (2001).

102. Goodman, S. M., Raheriarisena, M. & Jansa, S. A. A new species of *Eliurus* Milne Edwards, 1885 (Rodentia: Nesomyinae) from the Réserve Spéciale d’Ankarana, northern Madagascar. *Bonn. Zool. Beitr.* **56**(3), 133-149 (2009).

103. Carleton, M. D. & Goodman, S. M. A new species of the *Eliurus majori* complex (Rodentia: Muroidea: Nesomyidae) from south-central Madagascar, with remarks on emergent species groupings in the genus *Eliurus*. *Am. Mus. Novit.* **2007**, 1–21 (2007).

104. Carleton, M. D. Systematic studies of Madagascar’s endemic rodents (Muroidea: Nesomyinae): Revision of the genus *Eliurus*. *Am. Mus. Novit.* **3087**, 1–55 (1994).

105. Carleton, M. D. & Goodman, S. M. New taxa of nesomyine rodents (Muroidea: Muridae) from Madagascar’s northern highlands, with taxonomic comments on previously described forms. *Fieldiana Zool. Flor. Faunal Inventory Réserve Spéc. Anjanaharibe-Sud Madag. Ref. Elev. Var.* **90**, 163–200 (1998).

106. Rakotoarisoa, J.-E., Raheriarisena, M. & Goodman, S. M. A phylogeographic study of the endemic rodent *Eliurus carletoni* (Rodentia: Nesomyinae) in an ecological transition zone of northern Madagascar. *J. Hered.* **104**, 23–35 (2013).

107. Solano, E. *et al.* First karyotypic descriptions of Malagasy rodents (Nesomyinae, Muridae) reveal variation at multiple taxonomic levels. *J. Zool.* **285**, 110–118 (2011).

108. Goodman, S. M. & Soarimalala, V. A new species of *Macrotarsomys* (Rodentia: Muridae: Nesomyinae) from southwestern Madagascar. *Proc. Biol. Soc. Wash.* **118**, 450–464 (2005).

109. Goodman, S. M., Rakotondravony, D., Randriamanantsoa, H. N. & Rakotomalala-Razanahoera, M. A new species of rodent from the montane forest of central eastern Madagascar (Muridae: Nesomyinae: *Voalavo*). *Proc. Biol. Soc. Wash.* **118**, 863–873 (2005).

110. Rabarivola, C. Cytogenetic and molecular characteristics of a new species of sportive lemur from northern Madagascar. *Lemur News* **11**, 45–49 (2006).

111. Craul, M., Zimmermann, E., Rasoloharijaona, S., Randrianambinina, B. & Radespiel, U. Unexpected species diversity of Malagasy primates (*Lepilemur* spp.) in the same biogeographical zone: A morphological and molecular approach with the description of two new species. *BMC Evol. Biol.* **7**, 83 (2007).

112. Crowley, B. E. A refined chronology of prehistoric Madagascar and the demise of the megafauna. *Quat. Sci. Rev.* **29**, 2591–2603 (2010).

113. MacPhee, R. D. Morphology, adaptations, and relationships of Plesiorycteropus: And a diagnosis of a new order of eutherian mammals. *Bulletin of the AMNH* **220** (1994).

114. Mittermeier, R. A. *et al.* *Lemurs of madagascar*. (Conservation International, 2010).

115. Nowak, R. M. & Walker, E. P. *Walker’s Mammals of the World*. vol. 1 (JHU press, 1999).

116. Samonds, K. E. Late Pleistocene bat fossils from Anjohibe Cave, northwestern Madagascar. *Acta Chiropterologica* **9**, 39–65 (2007).

117. Burney, D. A. *et al.* A chronology for late prehistoric Madagascar. *J. Hum. Evol.* **47**, 25–63 (2004).

118. Godfrey, L. R., Jungers, W. L. & Burney, D. A. Subfossil lemurs of Madagascar. In *Cenozoic Mammals of Africa* 351–367 (2010).

119. Hoffmann, M. IUCN Red List of Threatened Species: *Palaeopropithecus ingens*. *IUCN Red List Threat. Species* (2008).

120. Perez, V. R. *et al.* Evidence of early butchery of giant lemurs in Madagascar. *J. Hum. Evol.* **49**, 722–742 (2005).

121. Quinn, A. & Wilson, D. E. *Daubentonia madagascariensis*. *Mamm. Species* **740**, 1–6 (2004).

122. Jungers, W. L. *et al.* The hands and feet of Archaeolemur: Metrical affinities and their functional significance. *J. Hum. Evol.* **49**, 36–55 (2005).

123. Muldoon, K. M. Paleoenvironment of Ankilitelo Cave (late Holocene, southwestern Madagascar): Implications for the extinction of giant lemurs. *J. Hum. Evol.* **58**, 338–352 (2010).

124. Yoder, A. D. *et al.* Single origin of Malagasy Carnivora from an African ancestor. *Nature* **421**, 734–737 (2003).

125. Goodman, S. M., Rasoloarison, R. M. & Ganzhorn, J. U. On the specific identification of subfossil *Cryptoprocta* (Mammalia, Carnivora) from Madagascar. *Zoosystema Paris* **26**, 129–143 (2004).

126. Boisserie, J.-R. IUCN Red List of Threatened Species: *Hippopotamus madagascariensis*. *IUCN Red List Threat. Species* (2016).

127. Goodman, S. M., Vasey, N. & Burney, D. A. Description of a new species of subfossil shrew tenrec (Afrosoricida: Tenrecidae: *Microgale*) from cave deposits in southeastern Madagascar. *Proc. Biol. Soc. Wash.* **120**, 367–376 (2007).

128. Turvey, S. T. *Holocene extinctions*. (OUP Oxford, 2009).

129. Crowley, B. E. & Samonds, K. E. Stable carbon isotope values confirm a recent increase in grasslands in northwestern Madagascar. *The Holocene* **23**, 1066–1073 (2013).

130. Douglass, K. *et al.* A critical review of radiocarbon dates clarifies the human settlement of Madagascar. *Quat. Sci. Rev.* **221**, 105878 (2019).

131. Godfrey, L. R. *et al.* A new interpretation of Madagascar’s megafaunal decline: The “Subsistence Shift Hypothesis”. *J. Hum. Evol.* **130**, 126–140 (2019).

132. Burney, D. A. Rates, patterns, and processes of landscape transformation and extinction in Madagascar. in *Extinctions in near time* 145–164 (Springer, 1999).

133. Burney, D. A. *et al.* New findings at Andrahomana Cave, southeastern Madagascar. *J. Cave Karst Stud.* **17**(1), 17-24 (2008).

134. Simon, E. L. Lemurs: Old and new. in *Natural change and human impact in Madagascar* (eds. Goodman, S. M. & Patterson, B. D.) 142–166 (Smithsonian Institution Press, 1997).

135. Tattersall, I. & Standing, H. F. A note on the age of the subfossil site of Ampasambazimba, Miarinarivo Province, Malagasy Republic. American Museum novitates **2520**, (1973).

136. Soarimalala, V. & Goodman, S. M. *Les Petits Mammifères de Madagascar*. (Association Vahatra, 2011).
