# Supplementary material for: The macroevolutionary impact of recent and imminent mammal extinctions on Madagascar
Source: Nat Commun. 2023 Jan 10;14:14. doi: 10.1038/s41467-022-35215-3 (PMC9832013; doi:10.1038/s41467-022-35215-3)
Supplement: Supplementary file 10 — Reporting Summary [file 41467_2022_35215_MOESM10_ESM.pdf]

nature portfolio

Corresponding author(s): Luis Valente

Last updated by author(s): Nov 11, 2022

Reporting Summary

Nature Portfolio wishes to improve the reproducibility of the work that we publish. This form provides structure for consistency and transparency in reporting. For further information on Nature Portfolio policies, see our Editorial Policies and the Editorial Policy Checklist.

Statistics

For all statistical analyses, confirm that the following items are present in the figure legend, table legend, main text, or Methods section.

n/a	Confirmed	
☐	☒	The exact sample size (*n*) for each experimental group/condition, given as a discrete number and unit of measurement
☒	☐	A statement on whether measurements were taken from distinct samples or whether the same sample was measured repeatedly
☒	☐	The statistical test(s) used AND whether they are one- or two-sided<br>*Only common tests should be described solely by name; describe more complex techniques in the Methods section.*
☒	☐	A description of all covariates tested
☒	☐	A description of any assumptions or corrections, such as tests of normality and adjustment for multiple comparisons
☐	☒	A full description of the statistical parameters including central tendency (e.g. means) or other basic estimates (e.g. regression coefficient) AND variation (e.g. standard deviation) or associated estimates of uncertainty (e.g. confidence intervals)
☒	☐	For null hypothesis testing, the test statistic (e.g. *F*, *t*, *r*) with confidence intervals, effect sizes, degrees of freedom and *P* value noted<br>*Give P values as exact values whenever suitable.*
☒	☐	For Bayesian analysis, information on the choice of priors and Markov chain Monte Carlo settings
☒	☐	For hierarchical and complex designs, identification of the appropriate level for tests and full reporting of outcomes
☒	☐	Estimates of effect sizes (e.g. Cohen's *d*, Pearson's *r*), indicating how they were calculated

Our web collection on statistics for biologists contains articles on many of the points above.

Software and code

Policy information about availability of computer code

Data collection	Excel v16.63<br>Figtree v1.4.4<br>R 4.2.1 and Rstudio 2022.02.3<br>R Code to extract data from posterior distribution of trees and scripts to run ERT analyses and reproduce figures available in https://github.com/luislvalente/madagascar and https://doi.org/10.5281/zenodo.7311466.
Data analysis	R 4.2.1 and Rstudio 2022.02.3<br>DAISIE R package 4.0.5 (https://zenodo.org/record/5708159#.YuPhyy8Rp9h)<br>Phytools 1.2-0 R package (http://www.phytools.org/)<br>Ape 5.6-2 package (https://cran.r-project.org/web/packages/ape/index.html)

For manuscripts utilizing custom algorithms or software that are central to the research but not yet described in published literature, software must be made available to editors and reviewers. We strongly encourage code deposition in a community repository (e.g. GitHub). See the Nature Portfolio guidelines for submitting code & software for further information.

Data

Policy information about availability of data

All manuscripts must include a data availability statement. This statement should provide the following information, where applicable:

- Accession codes, unique identifiers, or web links for publicly available datasets
- A description of any restrictions on data availability
- For clinical datasets or third party data, please ensure that the statement adheres to our policy

All the data that support the findings of this study are provided with this paper or have been deposited online. The Malagasy mammal checklist is available in the Supplementary Information. The phylogenetic trees and DAISIE objects are available in a Zenodo repository https://doi.org/10.5281/zenodo.7311466. The posterior distributions of phylogenetic trees are available in Mendeley Data https://doi.org/10.17632/wfs8k2pphd.1. We harmonized the taxonomy based on the Mammal Diversity Database available in Ref 67.

Human research participants

Policy information about studies involving human research participants and Sex and Gender in Research.

Reporting on sex and gender	NA
Population characteristics	NA
Recruitment	NA
Ethics oversight	NA

Note that full information on the approval of the study protocol must also be provided in the manuscript.

Field-specific reporting

Please select the one below that is the best fit for your research. If you are not sure, read the appropriate sections before making your selection.

☐ Life sciences ☐ Behavioural & social sciences ☒ Ecological, evolutionary & environmental sciences

For a reference copy of the document with all sections, see nature.com/documents/nr-reporting-summary-flat.pdf

Ecological, evolutionary & environmental sciences study design

All studies must disclose on these points even when the disclosure is negative.

Study description	We compiled phylogenetic and distribution data for all native species of mammals of Madagascar. We then gathered data on IUCN threat status for various years. For the extinct species we gathered information on the timing and causes of extinction. We extracted colonisation and branching times of these species from a dated phylogeny of mammals. We fitted the DAISIE model to these data to estimate rates of speciation, colonisation and extinction. Using these rates, we measured the evolutionary return time (ERT): for how long it would take to return to pre-human diversity; and how long it would take to return to current diversity if currently threatened species go extinct.
Research sample	We did not conduct experiments. Sample represents all non-marine species of mammal present on Madagascar before humans arrived. We sampled mammal species native to the island of Madagascar present in the Late Holocene (including present). The full species list and the references for the source of the data are provided in Supplementary Data S1.
Sampling strategy	We obtained phylogenetic data for all known species of mammals of the island of Madagascar.
Data collection	Data on Malagasy mammal species, IUCN status, phylogenetic data and updated mammal taxonomy were obtained from the literature and all sources are cited. Data was recorded by G.I.S, N.M.M., S.M.G., V.S., A.E.G. and L.V. using a computer.
Timing and spatial scale	Data was gathered between March 2020 and April 2022 from published sources. Spatial scale from which the data is taken is the island of Madagascar.
Data exclusions	No data were excluded.
Reproducibility	The script to extract and prepare the phylogenetic data for the analyses if available on https://github.com/luislvalente/madagascar. The likelihood and simulation analyses conducted in this study can be reproduced using examples provided in the R package DAISIE. Replication was done twice independently (N.M.M. and L.V.). All attempts at replication were successful.

Randomization	This is not relevant as we did not conduct experiments. There is only one mammal community on Madagascar and we studied the entire population.
Blinding	Blinding is not relevant, as we did not conduct experiments. There is only one mammal community on Madagascar and we studied the entire population.

Did the study involve field work? ☐ Yes ☒ No

Reporting for specific materials, systems and methods

We require information from authors about some types of materials, experimental systems and methods used in many studies. Here, indicate whether each material, system or method listed is relevant to your study. If you are not sure if a list item applies to your research, read the appropriate section before selecting a response.

Materials & experimental systems

n/a	Involved in the study
☒	☐ Antibodies
☒	☐ Eukaryotic cell lines
☒	☐ Palaeontology and archaeology
☒	☐ Animals and other organisms
☒	☐ Clinical data
☒	☐ Dual use research of concern

Methods

n/a	Involved in the study
☒	☐ ChIP-seq
☒	☐ Flow cytometry
☒	☐ MRI-based neuroimaging
